# Addressing the effectiveness of health literacy programs within the Gulf Corporation Council: an integrative review

**DOI:** 10.1093/heapro/daae062

**Published:** 2024-07-01

**Authors:** J Johnson, H Mohamed, T Lowe, F Khraim, C Wolsey, S Haque, A Al-Farsi, D Schnurman, N Chowdhury, M M H Raihan, T C Turin

**Affiliations:** Faculty of Nursing, Beal University Canada, 8 Main St, Sackville, Canada; National Center for Cancer Care and Research (NCCCR), Doha, Qatar; Faculty of Nursing, University of Calgary in Qatar, Doha, Qatar; Faculty of Nursing, Qatar University, Doha, Qatar; School of Nursing, University of Tasmania, Private bag 135, 7001 Hobart, Tasmania, Australia; Faculty of Nursing, University of Calgary, Doha, Qatar; Health and Wellness Education Department, Sidra Medicine Qatar, Doha, Qatar; Quality and Patient Safety, Sidra Medicine, Doha, Qatar; Department of Family Medicine, Cumming School of Medicine, University of Calgary, 3330 Hospital Dr, T2N 4N1, Calgary, Alberta, Canada; Department of Family Medicine, Cumming School of Medicine, University of Calgary, 3330 Hospital Dr, T2N 4N1, Calgary, Alberta, Canada; Department of Family Medicine, Cumming School of Medicine, University of Calgary, 3330 Hospital Dr, T2N 4N1, Calgary, Alberta, Canada

## Abstract

Health literacy is an increasingly required need to help individuals, families and communities manage their health and health conditions. It is linked with better self-adherence to treatments, use of resources, access to care and overall reduced costs in healthcare. In the Gulf Cooperation Council (GCC), which comprises Bahrain, Kuwait, Oman, Qatar, Saudi Arabia and the United Arab Emirates, various health literacy programs are implemented across states to address people’s unique and complex healthcare needs. This article aims to examine the current literature and assess the factors that influence the outcomes of health literacy programs within the GCC. An integrative review methodology has been conducted to pursue a comprehensive understanding of health literacy interventions in the GCC. This investigative approach was shaped by Whittemore and Knafl’s framework (2005), which includes problem identification, literature search, data evaluation, data analysis and presentation. The literature on the effectiveness of health literacy interventions and the factors that shape them are notably limited worldwide and within the GCC region. This integrative review addresses this knowledge gap and highlights the significance of key themes such as sessions, evaluation and improvement in shaping health literacy outcomes within the GCC region. Through this integrative review, the three main themes of sessions, evaluation and improvement were identified as influencing the outcomes of health literacy programs within the GCC.

**Contribution to Health Promotion**

- Health literacy is an important aspect of patient care.
- Understanding the multiple factors impacting health literacy is essential for developing and designing effective health literacy and health promotion interventions.
- Health literacy and health promotion interventions must be explored in various regions to understand and ensure optimal health.

## INTRODUCTION

Health literacy is required to help individuals, families and communities manage their health and health conditions (Elbashir *et al*., 2023; Johnson *et al*., 2022). Higher health literacy is linked with better self-adherence to treatments, use of resources, access to care and overall reduced costs in healthcare (Johnson *et al*., 2022). However, health literacy skills globally vary across regions, with reported ranges of 7–47% in developed nations (Johnson *et al*., 2022). Health literacy programs have emerged as strategies for enhancing skills and addressing the needs of patients, individuals and populations to minimize gaps. This strategy helps individuals and groups navigate complex healthcare systems and aids in positioning healthcare providers to be better engaged in providing patient-centred care, especially in managing disease and illness (Elmer *et al*., 2017). Health literacy programs address different topics and demographics and are tailored to accommodate the diverse needs of their populations. Knowing health literacy programs may vary across subjects, populations and regions, each needs to evaluate factors that influence its efficacy and outcomes (Elmer *et al*., 2017).

In the Gulf Cooperation Council (GCC), which comprises Bahrain, Kuwait, Oman, Qatar, Saudi Arabia and the United Arab Emirates, various health literacy programs are implemented across countries to address the unique and complex healthcare needs of people (Elbashir *et al*., 2023; Johnson *et al*., 2022; Khoja *et al*., 2017). While the correlation between health literacy and health outcomes is well known, there is less understanding of the efficacy of those interventions and the factors that may influence them (Elmer *et al*., 2017; Johnson *et al*., 2022). Understanding factors that influence the effectiveness of health literacy interventions is necessary to serve as a tool or model when developing future interventions in the region. This article aims to examine the current literature and assess the factors that influence the outcomes of health literacy programs within the GCC.

## METHOD

An integrative review can provide an array of detail to problems that may be complex, especially within the healthcare milieu. Therefore, an integrative review was chosen to investigate the phenomenon in question to help elucidate information related to health literacy interventions within the GCC. This integrative review was guided by Whittemore and Knafl’s (2005) framework, which includes problem identification, literature search, data evaluation, data analysis and presentation.

### Problem identification

The first step of Whittemore and Knafl’s (2005) framework for an integrative review is problem identification. With the initial scoping of the literature and discussion among the team members, we identified that it is essential to conduct a comprehensive search of the literature to understand various interventions and assess the factors affecting the outcomes of health literacy programs. Having clearly identified these problems, we developed our literature search and screening strategies, followed by appropriate analysis and reporting.

### Literature search

First, we identified the relevant databases that needed to be searched to ensure we did not miss any potentially eligible studies. We reviewed the other health literacy-related reviews to identify the relevant databases, including academic and grey literature. We also consulted with a systematic review expert to finalize the databases.

In the next step, we developed a search strategy to draw all the relevant articles from the databases. We identified the terms that bear similar meanings or are used as indexing terms (such as Medical Subject Headings) for different databases based on the three key terms ‘health literacy’, ‘Gulf Cooperation Council’ and ‘intervention’. After that, we developed the inclusion and exclusion criteria aligned with the study’s objectives to screen the articles abstracted from all the databases.

**Fig. 1:**
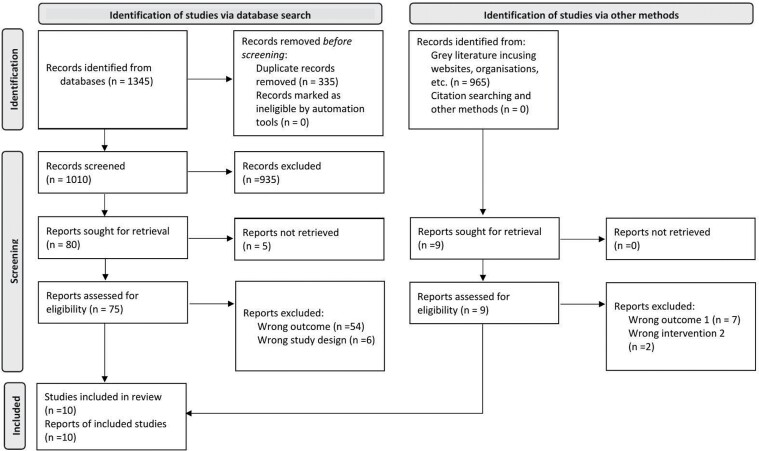
Flow diagram

We identified 1345 articles from the academic databases and 965 from the grey literature, resulting in 2310 articles. After duplicate removal, we had 1975 articles to screen. Two independent reviewers (M.R. and J.J.) first excluded the articles that did not meet the inclusion criteria based on the titles and abstracts of the articles. We used Rayyan QCRI software to facilitate this level of screening. The articles deemed eligible or inconclusive to exclude based on title/abstract did not move to the next screening level (*n* = 94). This time, both reviewers reviewed the articles’ full text and included only those meeting the inclusion criteria. In case of disagreement between the reviewers, both discussed with a third reviewer (N.C. or T.C.T.) and reached a consensus. Finally, we had 10 articles to analyse. See Figure 1 for flow chart and process.

### Data evaluation and extraction

Data evaluation was deemed unnecessary in this study as, according to our employed framework by Whittemore and Knafl (2005), evaluating the studies in an integrative review is not essential given the complex methodologies and findings drawn in this type of review. Also, our objective is broad and includes a summarization of a range of findings; thus, unlike a systematic review, it is not focused on synthesizing answers to a specific question from findings from studies with the same or related questions. Therefore, once we finalized the eligible articles, we moved to data synthesis directly following the data extraction. We used a predetermined data extraction template at this level.

### Data synthesis and presentation of the findings

We have coded the texts of the eligible studies using an open coding method. We looked for any information on the outcomes of health literacy assessments. Additionally, study characteristics such as location, sample size and others were collated for review. Once coding was complete, we identified the similar and contrasting codes to detect any emerging pattern. With the iterative process of reviewing the codes and the texts, we identified the eminent themes and provided a narrative description.

### Presentation of the results

#### Characteristics of the studies

This integrative review examines 10 research articles published between 2010 and 2022. These studies were conducted in the GCC region: Qatar (*n* = 1), the United Arab Emirates (*n* = 1) and the Kingdom of Saudi Arabia (*n* = 8). Articles are primary resources that consist of quantitative studies. There was one cross-sectional, one clustered experimental, one retrospective observational cohort pretest–posttest experimental, three quasi-experimental, two randomized trials and one cluster randomized control.

The cross-sectional study by Halawany *et al*. (2018) examined the effectiveness of oral health intervention in improving knowledge and self-reported oral health behaviour among primary school female children. Ba-Essa *et al*. (2015) assessed the impact of intensified self-monitoring of blood glucose with education on diabetes mellitus patients. Mahmoud *et al*. (2018) assessed the effects of a psychoeducational intervention program on an indicator of glycaemic control, and HR-QOL (health-related quality of life) was examined in type 2 diabetic patients. Sani *et al*. (2018) tested the effectiveness of the Jizan Integrated Lifestyle Education (JILSE) program on glycaemic control of diabetic patients. A cluster randomized trial by Alzaher *et al*. (2018) consisted of delivering a hand hygiene workshop intervention and reported its impact on reducing school children’s absenteeism due to upper respiratory tract infection. Mohammed *et al*. (2016) examined the efficacy of a smoking prevention program on smoking-related cognitions and behaviour for male adolescents. A retrospective observational cohort study by Rahhal *et al.*, (2021) compared the impact of clinical pharmacists and other healthcare providers’ education on patients’ adherence to post-percutaneous coronary intervention medication. Rabbani *et al.*, (2019) explored the effectiveness of a community-based educational intervention in promoting breast cancer screening and health behaviour changes. Bakarman *et al*. (2019) conducted a randomized interventional study to assess health-related quality of life among haemodialysis patients. Sharaf’s (2010) study assessed the impact of health education on the knowledge, behaviour and compliance of patients with hypertension, type 2 diabetes mellitus and coronary artery disease (Table 1). Sharaf’s (2010) study assessed the impact of health education on the knowledge, behaviour and compliance of patients with hypertension, type 2 diabetes mellitus and coronary artery disease. See Table 1 for all study characteristices and Table 2 for all health literacy initiavites. 

**Table 1:** Study characteristics

Author, year of publication and site	Objective	Target population	Study design	Population size	Key findings	Key conclusions
Alzaher *et al*. (2018)	To report the rate of episodes and days of absences due to upper respiratory infections (URIs) among schoolgirls over 5 weeks and to quantify their reduction after delivering a hand hygiene workshop intervention	Schoolgirls between the ages of 6 and 12 years. The study was conducted between January and March 2018	Cluster randomized trial	496 participants, 262 in the control group and 234 in the experimental group	The percentage of absence days was lower in the experimental group (Control group: 0.86% and 1.39% versus experimental group: 0.39% and 0.72%). Incidence rates of absence due to URIs were 0.54 and 1.02 in CG versus 0.24 and 0.51 in EG per 100 schoolgirls per day	There could be a further reduction in school absences if the dissemination of hand soap accompanied education
Riyadh, Saudi Arabia						
Ba-Essa *et al*. (2015)	To assess the impact of intensified SMBG with patient education on DM patients in the Eastern province of Saudi Arabia	All DM patients attending the Diabetes Center in Dammam Medical Complex	Quasi-single blind experimental design with pre- and post-comparison	60 patients (30 intervention group and 30 control group)	The intervention arm showed a significant reduction in the post-fasting glucose and HbA1c levels (*p* < 0.001) and a significant increase in glucose testing. The intervention group also improved knowledge, attitude and behaviour more than the control group.	This study observed that even with short duration, intensified monthly self-monitoring of blood glucose combined with education effectively improved glycaemic control, HDL levels, knowledge, attitude and practice scores in both insulin- and non-insulin-treated patients
Saudi Arabia						
Bakarman *et al*. (2019)	To assess health-related QOL among HD patients attending HD units in Jeddah, Saudi Arabia and to evaluate the effect of an educational program on HRQOL (health-related quality of life)	The study included all patients undergoing HD between January 2018 and May 2018	Randomized interventional study design	100 patients. 50 in a control group and 50 in an interventional group	- The overall QOL score improved in the patients who received the intervention and worsened in the control group. - The educational program had a significant positive impact on all health-related QOL parameters	Providing periodic counselling at regular intervals will improve the QOL of HD patients
Jeddah, Saudi Arabia						
Halawany *et al*. (2018)	To examine the effectiveness of oral health intervention on the improvement in knowledge and self-reported oral health behaviour among 6- to 8-year-old female primary school children in Riyadh, Saudi Arabia	Girls in primary schools who are 6- to 8-year-olds (first, second and third graders)	Cross-sectional study	1661 girls from primary schools	There was a significant increase in the level of knowledge by 11.24% and self-reported behaviour by 25% after intervention (*p* < 0.001)	The results of this study showed that an easy-to-organize and inexpensive school-based intervention could, on a short-term basis, be effective in improving children’s knowledge and self-reported oral health behaviour
Riyadh, Saudi Arabia						
Mahmoud *et al*. (2018)	To assess the effects of a psychoeducational intervention program on an indicator of glycaemic control and HRQoL (health-related quality of life) among type 2 diabetic patients	Outpatients with type 2 diabetes	A quasi-experimental	99 patients	After the intervention, there was a statistically significant reduction in the mean value of HbA1c from 9.8 to 7.7 (*p* < 0.001), and there was a significant improvement in the mean scores of the following health-related QOL scales	The application of such psychoeducational intervention programs can help improve HbA1c levels and quality of life for patients with DM (diabetes mellitus)
Jizan City, Saudi Arabia						
Mohammed *et al*. (2016)	To examine the efficacy of a smoking prevention program that aimed to address smoking-related cognitions and smoking behaviour among Saudi adolescents aged 13–15	Male Saudi adolescents aged 13–15	Randomized controlled trial	The experimental group (*N* = 698), the control group (*N* = 683)	Post-interventions results revealed that the experimental group had a significantly more negative attitude towards smoking, stronger social norms against smoking, higher self-efficacy towards non-smoking, more action planning to remain a non-smoker and lower intentions to smoke in the future	The prevention program reinforced non-smoking cognitions and non-smoking behaviour.
Taif province, Saudia Arabia						
Rabbani *et al.*, (2019)	To explore whether a community-based educational intervention is an effective approach for improving the awareness of BC and its screening procedures and promoting health behaviour change	All adult females living in the study setting were considered for the study	A one-group pretest–posttest experimental design	250 women	After the educational intervention, statistically significant improvement was observed in all the BC knowledge domains. Findings revealed that the intervention proved more beneficial to women without formal education. - Post-intervention, the women were more positive towards medical help-seeking and acknowledged the fact that BC diagnosed early is more treatable	Community-based education has proved effective in raising awareness of this study population.
Ras al Khaimah (RAK), UAE						
Rahhal *et al.*, (2021)	To assess the impact of education provided by clinical pharmacists compared with other healthcare providers on adherence to post-PCI medications and clinical outcomes among patients with ST-elevation myocardial infarction (STEMI)	All patients admitted with STEMI to Heart Hospital in Qatar between 1 January 2016 and 31 December 2018	Retrospective observational cohort study	1334 adult patients. Patients who were educated by clinical pharmacists were included in the intervention group, and patients who other healthcare providers educated were included in the control group.	Only 26% of the study population were educated by clinical pharmacists, while other healthcare providers educated others. Adherence to post-PCI medications was significantly better in patients whom clinical pharmacists educated	Patients’ adherence to post-PCI medications significantly improved with clinical pharmacists’ counselling
Qatar						
Sani *et al*. (2018)	To test the effectiveness, feasibility and acceptability of the Jizan Integrated Lifestyle Education (JILSE) program	Newly diagnosed diabetes mellitus patients	A quasi-experimental two-group pre- and post-evaluation study design	200 patients	There was a statistically significant reduction in the intervention group compared to the control group. Reduction in all parameters. However, smoking habits and physical exercise were not statistically significant (*p* > 0.05). The diabetic knowledge level was significantly improved after the JILSE program	Diabetic patient peer group networks with periodic health education messages and motivation from specialists can decrease the blood glucose level, systolic and diastolic blood pressure and BMI among diabetes type 2 patients
Jizan City, Saudi Arabia						
Sharaf (2010)	To assess the impact of health education on the health knowledge and behaviour of patients of three chronic diseases (hypertension, type 2 diabetes mellitus and coronary artery disease)	The target population included patients with chronic diseases and patients visiting for other complaints.	Clustered experimental study	The first 100 patients who visited each PHC were recruited. 1011 completed the study intervention.	- At baseline, chronic disease patients had healthier diets and exercised more than patients with other complaints. - Among chronic disease patients, significant improvements in smoking, diet and exercise habits were observed at the end-line survey compared to baseline. - These changes persisted and, in some cases, enhanced after controlling for age, sex, marital status and education	Designed patient education programs directed at patients with chronic diseases would have a much more significant effect
Al Qassim province, Saudi Arabia						

## FINDINGS

This integrative review aimed to assess the factors influencing the outcomes of health literacy programs within the GCC countries. Three main themes were identified while reviewing information in the literature: sessions, evaluation and improvement.

### Sessions

Educational programs that enhance health literacy in the GCC were tailored in multiple sessions. These sessions were delivered individually or in a group manner utilizing strategies such as didactic presentations, peer support and interactive discussions, among others. Depending on the program, most sessions lasted from about 30 min to 1 h over weeks to months. It is important to highlight that these sessions were age-based, provided supportive materials to the clients and were culturally oriented.

The participants’ ages in these health literacy educational sessions ranged from 6 to 75. These sessions were developed with the participants’ age groups in mind to achieve the main objective of enhancing health literacy. For instance, Halawany and colleagues (2018) used animated videos to improve 6-year-old students’ knowledge and oral hygiene behaviour. Similarly, Alzaher *et al.*’s (2018) study used puzzle games and cartoon princess pictures to teach schoolgirls the importance of hand washing. Video peer sessions led by participants of the same age group were another method Mohammed *et al*. (2016) used to teach male adolescents about smoking prevention. In contrast, sessions designed for adult participants were based on didactic lecturing and discussions (Bakarman *et al*., 2019; Rahhal *et al.*, 2021).

In addition to age, these sessions were equipped with various educational materials distributed during or after the sessions concluded. Alzaher *et al*. (2018) and Ba-Essa *et al*. (2015) provided participants with posters, leaflets, transcribed pamphlets and video films as audiovisual aids to remind them of the given information.

In the GCC region, cultural beliefs and values are highly associated with compliance with prescribed regimens. Therefore, when developing these educational sessions, developers utilized as many culturally appropriate resources as possible. In Sharaf’s (2010) study, investigators noted that smoking was becoming a part of the culture in Saudi Arabia. Hence, this was considered when assessing the impact of health education on the health knowledge and behaviour of patients with chronic diseases. Also, in the study by Mohammed *et al*. (2016), researchers adopted a Dutch smoking prevention program but adapted it to meet the cultural norms consistent with Saudi culture. The cultural aspect was maintained constantly when these educational sessions were developed by Alzaher *et al*. (2018), and the suitability of the sessions to the local cultural conditions was tested by Sani *et al*. (2018) before delivering their education. In short, the GCC has a unique culture that impacts health decisions; hence, researchers maintained this aspect when considering any educational session they delivered to clients.

### Evaluation

Evaluation is required when delivering health literacy programs to assess the attendees’ knowledge and understanding of desired outcomes and program effectiveness. Investigators who conducted these programs from a health literacy lens followed up with their participants, while others compared pre- and post-interventions to check compliance. Alzaher *et al*. (2018) followed up with participants after 5 weeks of attending the diabetic management program to examine whether participants maintained the suggested lifestyle changes. These authors found that this period was not too short to assess compliance properly: the same was noted by Bakarman *et al*., 2019. Other investigators followed up with participants after 6 months of interventions. Mohammed *et al*. (2016) examined the effects of interventions on socio-cognitive factors and behavioural effects, which was congruent with Sharaf’s (2010) study, which also noted that with more time, there is more compliance with diabetic regimens. According to Ba-Essa *et al*.’s (2015) study, which looked at glucose self-monitoring education in diabetes mellitus patients in Saudi Arabia, the authors conducted regular monthly follow-ups of patients with diabetes for 4 months straight to ensure compliance was maintained.

Pre- and post-intervention assessment is another form of evaluation to monitor the effectiveness of health literacy programs. Investigators of these studies used surveys to compare pre- and post-effectiveness in their participants (Ba-Essa *et al*., 2015; Mahmoud *et al*., 2018). Halawany and colleagues (2018) distributed questionnaires for pre-diabetic training 6 weeks after post-delivering their program to evaluate the level of improvement of knowledge and self-reported behaviour of their participants in oral health. Comparing pre- and post-surveys was noted to be a common strategy in assessing the effectiveness of health literacy in diabetic management programs (e.g. Bakarman *et al*., 2019;; Sani *et al*., 2018).

### Improvement

Examining the effectiveness of health literacy programs can increase the knowledge enhancement of recipients, modify behaviours, improve compliance with comorbidity regimens and improve health-related quality of life. Increased knowledge of participants’ health literacy levels is evidence of the effectiveness of these programs in the GCC. In a study by Rabbani *et al.*, (2019), participants showed statistically significant improvement in their knowledge, and interventions were more beneficial to the women without formal education (*p* = 0.001). Also, participants enrolled in diabetes health literacy programs significantly increased their knowledge (Ba-Essa *et al*., 2015; Mahmoud *et al*., 2018; Sani *et al*., 2018). Increased knowledge demonstrated by participants is a key factor influencing the effectiveness of health literacy programs (*p* = 0.001).

In addition to knowledge, behavioural change is another component that measures the success of these programs. Investigators such as Halawany *et al*. (2018) aimed to examine self-reported oral health behaviour among schoolchildren and noted a 25% significant improvement in their health-related behavioural interventions. Like Ba-Essa *et al.*’s (2015) study, they found more significant improvement in the attitude and behaviour of participants in the experimental group compared to the control group. Moreover, Sharaf’s (2010) study assessed the knowledge and behaviour of patients with comorbidities (*n* − 1011). He found significant improvements in smoking, diet and exercise habits in the study participants once the program was delivered. On the other hand, smoking habits and physical exercise were not statistically significant (*p* > 0.05) in a similar study conducted by Sani *et al*. (2018). Collectively, behavioural change is a common indicator in these health literacy interventions.

Patients with comorbidity are anticipated to benefit from health literacy programs, thereby modifying their health decisions and complying with recommended regimens. It was noted that patient compliance with the comorbidity regimen is a valued parameter in improving the effectiveness of health literacy programs. In the study by Rahhal *et al.*, (2021), investigators assessed compliance with the regimen of adult patients (*n* = 1334) who underwent percutaneous coronary intervention (PCI) and received clinical pharmacists’ educational interventions during discharge from the hospital. These investigators found significant adherence to post-PCI medication. Another study concluded that health education for participants with chronic diseases significantly improved compliance with their primary doctors’ advice regarding smoking, diet and exercise (Sharaf, 2010).

Besides compliance with comorbidity regimens, enhancing health-related quality of life (HR-QOL) is another desirable goal noted by health literacy programs. According to Bakarman *et al*. (2019) study, an overall increase in the scores of HR-QOL for patients with haemodialysis significantly improved after educational interventions compared to those in the control group. It was also noted that there was a significant improvement in the mean scores of HR-QOL scales for patients with type 2 diabetes mellitus in Mahmoud *et al*. (2018) study. Ba-Essa *et al*. (2015) study worked to ensure their health literacy program was aimed at improving overall health status and quality of life for patients with type 1 and 2 diabetes mellitus.

## DISCUSSION

There is scant literature about the efficacy of health literacy interventions worldwide and the factors that influence them, specifically within the GCC region. This integrative review closes this gap and identifies the themes of sessions, evaluation and improvement as factors influencing health literacy outcomes in the GCC countries.

Under the session’s theme, interventions associated with increased health literacy were provided using multiple sessions tailored to age and culture. A systematic review by Walters *et al*. (2020) of 22 studies similarly found that the majority of health literacy interventions were provided in multiple sessions and also had variations in session frequency and duration, with session lengths ranging from 40 min to all day at different frequencies (e.g. twice a week, weekly, every 2 weeks and monthly) for a total duration of 2 weeks to 12 months (Walters *et al*., 2020). Mirroring the findings from this review, interventions were delivered individually or in groups, with small group sessions being the more common delivery method (Walters *et al*., 2020). For adults, various strategies were likewise used, including educational sessions, text or social media messages, leaflets, psychosocial support, counselling and animation videos (Walters *et al*., 2020). Interventions incorporated into the school setting illustrate a crucial age-based approach to promoting health literacy among young people (World Health Organization, 2017). Effective intervention strategies used with children and adolescents utilize a practical, ‘hands-on’ approach, such as mindfulness or cooking activities, performing skits, using social media and incorporating peer models or peer support. Like the peer-led smoking prevention groups in the Mohammed *et al*. (2016) study, they are holistic by engaging schools, families and communities (Pleasant *et al*., 2019; Smith *et al*., 2021). The cultural adaptations to health education programs described above are supported by multiple studies illustrating how culturally sensitive considerations help to promote health literacy (Bader *et al*., 2022; Li *et al*., 2023; Tucker *et al*., 2019). Such considerations include having educational materials available in the relevant languages (Bader *et al*., 2022; Li *et al*., 2023) and in audiovisual format (Li *et al*., 2023) and incorporating influential community figures, such as religious leaders (Tucker *et al*., 2019), into the delivery of health literacy interventions. Further research is needed to explore any difference in efficacy between single vs. multiple-session health intervention delivery and associated outcomes.

The evaluation of health literacy programs is important to determine the effectiveness of the implemented interventions. This integrative review found that evaluation of programs in the GCC occurred through follow-up with study participants to assess for compliance and through pre- and post-intervention surveys to assess participant knowledge and behaviour. Such evaluation measures (follow-up for compliance and pre–post-intervention assessment) can provide evidence linking a specific intervention with health literacy outcomes (e.g. increased or decreased) if any change occurs. Follow-up occurred several (4–5) months after the intervention for the studies in this review, whereas follow-up in the Bader *et al*. (2022) review ranged from 2 weeks to 1 year. Nutbeam *et al*. (2018) reviewed the literature and found six studies that utilized a pre–post-study design to assess health literacy interventions, further supporting the importance of evaluation. Comparison assessments were also made in the Walters *et al*. (2020) systematic review, where all 22 of the included studies contained a pre–post measure of health literacy based on subjective self-reports, objective measures or validated instruments, such as the newest vital sign (NVS), test of functional health literacy in adults / short test of functional health literacy in adults (TOFHLA/STOFHLA) and the Health Literacy Questionnaire (HLQ). Post-assessment occurred as early as the same day of the intervention up through 12 months following the intervention (Walters *et al*., 2020).

The final improvement theme can be demonstrated through increased knowledge, behaviour change, treatment regimen compliance and health-related quality of life improvement. Similar indicators of improvement are evident in numerous studies. In the systematic review by Walters *et al*. (2020), 15 of the 22 identified studies (*n* = 10 181) demonstrated improvements in health literacy associated with implemented interventions, with a statistically significant increase in 12 studies. Health literacy outcome measures were not always clearly defined and ranged from subjective self-reported information to objective data and validated tools. Greater standardization of outcome measures will help optimize health literacy assessment (Bader *et al*., 2022). Seven out of eight studies with behavioural measures demonstrated significant improvements in behavioural outcomes following health interventions. The behavioural effects included smoking prevention, nutrition, physical activity, cancer screening, lifestyle, self-care and cardiovascular health (Walters *et al*., 2020). Regarding compliance with comorbidity regimens, an educational intervention consisting of three group workshops significantly affected health literacy, self-efficacy and self-care behaviours for patients with heart failure both at the time of the intervention and during follow-up in 3 months (Walters *et al*., 2020). Stellefson *et al*. (2019) found increased health-related quality-of-life scores significantly associated with higher health literacy levels in individuals diagnosed with chronic obstructive pulmonary disease.

## LIMITATIONS

Limitations of this integrative review highlight the need for further health literacy studies in all regions of the GCC. Most studies included in the review were from Saudi Arabia, with no study included in the review from Bahrain or Oman. Further studies assessing health literacy interventions directly are needed in the GCC region.

## CONCLUSION

The efficacy and outcomes of health literacy program interventions must be evaluated within the context of the region and population. Through this integrative review, the three main themes of sessions, evaluation and improvement were identified as influencing the outcomes of health literacy programs within the GCC. Ten studies were assessed for characteristics, study findings and evaluation of efficacy. These studies deployed various interventions and included the development of culturally appropriate materials or modifying adopted programs. Identifying these core themes can serve as a model for developing future health literacy interventions in the GCC region.

**Table 2:** Description of health literacy initiatives in included studies

Author, year of publication and site	Content of health literacy	Health literacy activities	Settings	Description of the initiative	Level of community engagement	Partners from knowledge-users
Alzaher *et al*. (2018)	Common infections in schools, methods of transmission and a handwashing procedure using soap and water, including when to wash hands	- Baseline questionnaires completed by participants - After 1 week, participants attended a 1-h Arabic handwashing workshop	In 4 public primary girls’ schools in the city of Riyadh, Saudi Arabia	- Workshops included a 6-min video-clip - Short, interactive lecture - Puzzle games related to hand hygiene were distributed among schoolgirls - Posters with cartoon princesses’ pictures promoting hand washing were also distributed in the schools	The principal investigator conducted the workshops	Participants’ parents fill out the questionnaire pre and post-intervention
Saudi Arabia						
Bakarman *et al*. (2019)	- Introduction to renal disease and HD - Dietary advice - Care of vascular access site - Coping strategies	- Demographic data and laboratory results were recorded - A survey of QOL were obtained pre and post-educational sessions - Individualized educational sessions by researchers	At 4 different governmental hospital dialysis units	Individualized health educational sessions were delivered by the researchers twice a week for a total duration of 8 weeks, and each session lasted for approximately 30 min	A dietician collaborated to highlight the right number of calories, protein quantity and serving sizes, including adequate amounts of sodium, potassium and phosphorus, in verbal and written format	NIL
Saudi Arabia						
Ba-Essa *et al*. (2015)	- About the disease, types, risk factors, symptoms, seriousness, management, self-care, food items of low carbohydrate and high fibre content, exercise and self-monitoring of blood glucose	- The program was implemented for 4 months (baseline) and 3 monthly follow-up visits - All patients in the intervention group had a one-on-one initial education session followed by three more sessions every month during follow-up - The intervention was monitored and evaluated using a combination of questionnaire interviews, observation of performance and laboratory investigations	In the Diabetes Center in Dammam Medical Complex	Patients received posters, leaflets and video films as audiovisual aids, as well as pamphlets to remind them of the education session.	Researchers and health educators conducted the education sessions	NIL
Saudi Arabia						
Halawany *et al*. (2018)	Brushing teeth twice per day, healthy diet and regular dental visit twice per year	- The children’s level of knowledge was assessed by a self-administered questionnaire divided into oral health knowledge and self-reported oral health behaviour - The questionnaires were distributed before and 6 weeks after implementation of the oral health educational program	Government schools from low to middle socioeconomic status were chosen in Riyadh city.	The oral health education program included four 4-min animation videos, a lecture presentation and four educational corners	19 undergraduate dental students, under the supervision of 6 faculty members of the Department of Periodontics and Community Dentistry conducted the program	NIL
Riyadh, Saudi Arabia						
Mahmoud *et al*. (2018)	A diabetes overview and its complications, self-care, medications and their side effects, lifestyle modification, clarification of myths and misconceptions and coping skills for living with diabetes	- Formal interviews were conducted to collect demographic data, DM history and health-related QOL. - Psychoeducational sessions delivered - HRQoL and HbA1c were reassessed to determine the effects of the program intervention on them	In four primary health care centres (PHCCs) in Jizan from August 2016 through March 2017	The psychoeducational program was based on interactive educational methods and techniques involving counselling, demonstration, group discussion and vignettes. - Education sessions were held at the chronic disease clinic for 3 h weekly for 4 weeks, and then participants were followed for 5 months	One psycho-educator and one nurse held the educational class for each group	NIL
Jazan, Saudi Arabia						
Mohammed *et al*. (2016)	NIL	- Baselines were collected for demographic data and smoking behaviour - A 5-week program was implemented - Follow-up data were collected after 6 months	19 male secondary schools in Taif province	The program used a video peer-led approach, implying that the main theme was introduced on video by youngsters, followed by group work and active learning The intervention consisted of five lessons; each lesson took 45 min	Trained school healthcare workers guided the intervention program, while peer leaders whom group members selected were trained on how to lead the discussion and how to make and present a summary	NIL
Saudi Arabia						
Rabbani *et al.*, (2019)	The session included general information on BC, BC epidemiology, breast anatomy, BC risk factors, signs and symptoms, the importance of early detection, screening procedures, breast self-examination (BSE) and mammography, the role of BSE and mammography in early diagnosis of BC and treatment options for BC.	Baseline knowledge (pre–test) regarding BC was assessed using the study questionnaire. - Educational intervention in BC was administered. - The impact of educational intervention was evaluated with a post-test questionnaire after 4 weeks.	- Community-based study. - No specific setting was reported	Not reported	NIL	NIL
United Arab Emirates						
Rahhal *et al.*, (2021)	NIL	Patients who get discharged in the morning shift during the weekdays were educated by clinical pharmacists after collecting their discharge medications. Conversely, those discharged on the weekends and evening shifts during the weekdays are educated by physicians, outpatient hospital pharmacists, or nurses after collecting their discharge medications	Heart Hospital is a specialized tertiary cardiology hospital that is part of Hamad Medical Corporation (HMC)	Data on educational interventions were collected from the HMC electronic medical record system using Cerner	Physicians, outpatient hospital pharmacists and nurses gave education to the control group	NIL
Qatar						
Sani *et al*. (2018)	The JILSE program educates the dynamics of a disease process, risk factors and complications, the importance of regular check-ups, medication and monitoring, diabetic diet, dietary approaches to stop hypertension (DASH diet), the importance of regular physical activity/exercise, quit smoking and Khat, positive attitude, stress management, the regular use of pedometer, automatic BP apparatus, blood glucose monitor, yoga and use of personal medical diary. Interesting practical sessions with hands-on experiences regarding purchasing options, cooking techniques, regular use of instruments, management of complications and self-care were included	- Primary assessment conducted for demographic data and laboratory tests - The Michigan Diabetes Knowledge Test (MDKT) questionnaire was used pre- and post-intervention - Participants exposed to the JILSE program	Jizan Diabetic Center	- Separate whats app groups exclusively for each diabetes cluster (DC) were formed. - Each DC worked as a peer group and interacted with each other - Monthly meetings were organized at the Diabetic Center	Specialist doctors developed the program and were available in each session as experts to provide scientific input on topics selected by the Diabetes Circle	NIL
Saudi Arabia						
Sharaf (2010)	Enhanced health education program on smoking, diet and exercise	A baseline survey was conducted to establish the prevalence of common risk factors of chronic diseases. - Then 6-month-long intervention. - End-line survey conducted at the end of the intervention to evaluate the educational sessions.	In selected 15 PHC centres	The patients received health education at the PHC centres from their doctors and health educators, who had received intensive refresher training in health education techniques and interpersonal communications skills. The training’s objective was to improve the healthcare providers’ knowledge and skills regarding health education.	Doctors, health educators and other paramedical workers delivered the educational sessions for patients. Education sessions in the PHC centres were also organized with the help of medical students from Qassim University	NIL
Al Qassim, Saudi Arabia						

## References

[CIT0001] Alzaher, A. A., Almudarra, S. S., Mustafa, M. H. and Gosadi, I. M. (2018) The importance of hand hygiene education on primary schoolgirls’ absence due to upper respiratory infections in Saudi Arabia: a cluster randomized controlled trial. Saudi Medical Journal, 39, 1044–1049. 30284589 10.15537/smj.2018.10.23344 PMC6201029

[CIT0002] Bader, M., Zheng, L., Rao, D., Shiyanbola, O., Myers, L., Davis, T. et al. (2022) Towards a more patient-centred clinical trial process: a systematic review of interventions incorporating health literacy best practices. Contemporary Clinical Trials, 116, 106733. 35301134 10.1016/j.cct.2022.106733 PMC9196949

[CIT0003] Ba-Essa, E. M., Mobarak, E. I., Alghamdi, A. and Al-Daghri, N. M. (2015) Intensified glucose self-monitoring with education in Saudi DM patients. International Journal of Clinical and Experimental Medicine, 8, 19374–19380. 26770578 PMC4694478

[CIT0004] Bakarman, M. A., Felimban, M. K., Atta, M. M. and Butt, N. S. (2019) The effect of an educational program on quality of life in patients undergoing hemodialysis in western Saudi Arabia. Saudi Medical Journal, 40, 66–71. 30617383 10.15537/smj.2019.1.23401 PMC6452607

[CIT0005] Elbashir, M., ElHajj, M. S., Rainkie, D., Kheir, N., Hamou, F., Abdulrhim, S. et al. (2023) Evaluation of health literacy levels and associated factors among patients with acute coronary syndrome and heart failure in Qatar. Patient Preference and Adherence, 17, 89–105. 36642998 10.2147/PPA.S385246 PMC9835006

[CIT0006] Elmer, S., Bridgman, H., Williams, A., Bird, M. L., Murray, S., Jones, R. et al. (2017) Evaluation of a health literacy program for chronic conditions. Health Literacy Research and Practice, 1, e100–e108. 31294255 10.3928/24748307-20170523-01 PMC6607796

[CIT0007] Halawany, H. S., Al Badr, A., Al Sadhan, S., Al Balkhi, M., Al-Maflehi, N., Abraham, N. B. et al. (2018) Effectiveness of oral health education intervention among female primary school children in Riyadh, Saudi Arabia. The Saudi Dental Journal, 30, 190–196. 29942102 10.1016/j.sdentj.2018.04.001 PMC6011217

[CIT0008] Johnson, J., Khraim, F., Wolsey, C., Chowdhury, N., Raihan, M. M. H. and Turin, T. C. (2022) Addressing health literacy in the Gulf Cooperation Council (GCC) countries: an integrative review protocol to summarize the health literacy landscape. Middle East Journal of Nursing, 16, 33–41.

[CIT0009] Khoja, T., Rawaf, S., Qidwai, W., Rawaf, D., Nanji, K. and Hamad, A. (2017) Health care in Gulf Cooperation Council countries: a review of challenges and opportunities. Cureus, 9, e1586. 29062618 10.7759/cureus.1586 PMC5650259

[CIT0010] Li, B., Huang, Y., Ling, C., Jiao, F., Fu, H. and Deng, R. (2023) The effect of community-based health education programs on health literacy in severely impoverished counties in Southwestern China: results from a quasi-experimental design. Frontiers in Public Health, 10, 1088934. 36703836 10.3389/fpubh.2022.1088934 PMC9871388

[CIT0012] Mahmoud, S. S., Mahdy, M. H. E., Mahfouz, M. S., Nada, I. S., Aqeeli, A. A., Al Darbi, M. A. et al. (2018) Effects of a psychoeducational program on hemoglobin A1c level and health-related quality of life in patients with type 2 diabetes mellitus, Jazan, Saudi Arabia. Biomed Research International, doi: 10.1155/2018/6915467 PMC5976993 29862283

[CIT0011] Mohammed, M., Eggers, S. M., Alotaiby, F. F., de Vries, N. and de Vries, H. (2016) Effects of a randomized controlled trial to assess the six-month effects of a school-based smoking prevention program in Saudi Arabia. Preventive Medicine, 90, 100–106. 27386742 10.1016/j.ypmed.2016.06.032

[CIT0013] Nutbeam, D., McGill, B. and Premkumar, P. (2018) Improving health literacy in community populations: a review of progress. Health Promotion International, 33, 901–911. 28369557 10.1093/heapro/dax015

[CIT0014] Pleasant, A., Griffin, K., Maish, C., O’Leary, C. and Carmona, R. (2019) Health literacy interventions for children or adolescents: an overview and insights into practical applications. In Orkan, O., Bauer, U., Levin-Zamir, D., Pinheiro, P. and Sorensen, K. (eds), International Handbook of Health Literacy: Research, Practice, and Policy across the Lifespan. Policy Press, Bristol, pp. 307–322. doi: 10.51952/9781447344520ch020

[CIT0015] Rabbani, S. A., Al Marzooqi, A. M., Srouji, A. E., Hamad, E. A. and Mahtab, A. (2019) Impact of community-based educational intervention on breast cancer and its screening awareness among Arab women in the United Arab Emirates. Clinical Epidemiology and Global Health, 7, 600–605.

[CIT0016] Rahhal, A., Mahfouz, A., Al‐Amri, M., Aljundi, A., Khir, F., Hamid, Y. et al. (2021) Impact of discharge education by clinical pharmacists on patients’ adherence to post‐percutaneous coronary intervention medications: a retrospective cohort study using real‐world data. Journal of the American College of Clinical Pharmacy, 4, 303–310.

[CIT0017] Sani, M., Makeen, A., Albasheer, O., Solan, Y. and Mahfouz, M. S. (2018) Effect of telemedicine messages integrated with peer group support on glycemic control in people with type 2 diabetes, Kingdom of Saudi Arabia. International Journal of Diabetes in Developing Countries, 38, 495–501.

[CIT0018] Sharaf, F. (2010) Impact of health education on compliance among patients of chronic diseases in Al Qassim, Saudi Arabia. International Journal of Health Sciences, 4, 139–148. 21475552 PMC3068830

[CIT0019] Smith, C., Goss, H. R., Issartel, J. and Belton, S. (2021) Health literacy in schools? A systematic review of health-related interventions aimed at disadvantaged adolescents. Children (Basel, Switzerland), 8, 176. 33668861 10.3390/children8030176 PMC7996245

[CIT0020] Stellefson, M., Paige, S. R., Alber, J. M., Chaney, B. H., Chaney, D., Apperson, A. et al. (2019) Association between health literacy, electronic health literacy, disease-specific knowledge, and health-related quality of life among adults with chronic obstructive pulmonary disease: cross-sectional study. Journal of Medical Internet Research, 21, e12165. 31172962 10.2196/12165 PMC6592488

[CIT0021] Tucker, C., Kang, S., Ukonu, N., Linn, G., DiSangro, C., Arthur, T. et al. (2019) A culturally sensitive church-based health-smart intervention for increasing health literacy and health-promoting behaviours among black adult churchgoers. Journal of Health Care for the Poor and Underserved, 30, 80–101. 30827971 10.1353/hpu.2019.0009

[CIT0022] Walters, R., Leslie, S. J., Polson, R., Cusack, T. and Gorely, T. (2020) Establishing the efficacy of interventions to improve health literacy and health behaviours: a systematic review. BMC Public Health, 20, 1040. 32605608 10.1186/s12889-020-08991-0 PMC7329558

[CIT0023] Whittemore, R. and Knafl, K. (2005) The integrative review: updated methodology. Journal of Advanced Nursing, 52, 546–553. 16268861 10.1111/j.1365-2648.2005.03621.x

[CIT0024] World Health Organization. (2017) Shanghai declaration on promoting health in the 2030 Agenda for Sustainable Development. Health Promotion International, 32, 7–8. 28180270 10.1093/heapro/daw103

